# Microbial community structure dynamics of invasive bullfrog with meningitis-like infectious disease

**DOI:** 10.3389/fmicb.2023.1126195

**Published:** 2023-03-13

**Authors:** Wengang Li, Guangwei Fan, Ke Sun, Jingru Liu, Jinyan Liu, Yu Wang, En Li, Xiaobing Wu, Liang Shen, Tao Pan

**Affiliations:** ^1^College of Life Sciences, Anhui Normal University, Wuhu, Anhui, China; ^2^Anhui Provincial Key Laboratory of Conservation and Exploitation of Biological Resources, Wuhu, China

**Keywords:** bullfrogs, changed, meningitis-like infectious disease, microbial community, *Elizabethkingia*

## Abstract

Meningitis-like infectious disease (MID) (also known as frog cataract and torticollis) is a disease prone to occur in amphibians and reptiles. It is highly contagious and has a high mortality rate. In this study, we sampled and sequenced microbiomes from oral and intestinal samples of five normal and five diseased bullfrogs. The analysis found that the richness, uniformity, and abundance of the microbial community of the diseased bullfrogs were significantly higher than those of the normal bullfrogs in both the oral cavity and the gut. In the diseased group, the abundance of *Elizabethkingia* significantly increased and that of *Lactococcus* significantly decreased. It showed that the structure of the microbial community had changed a lot in diseased frogs. After the pathogenic bacteria infected the body, it might be make the decline in the immune function of the body declined, and resulting in some conditional pathogenic bacteria in the water body further infecting the body. As a result, the richness and composition of the microbial community significantly changed. This study can provide a theoretical basis for the control of MID of bullfrogs.

## Introduction

### Benefits and challenges of bullfrog farming

**Figure 1:**
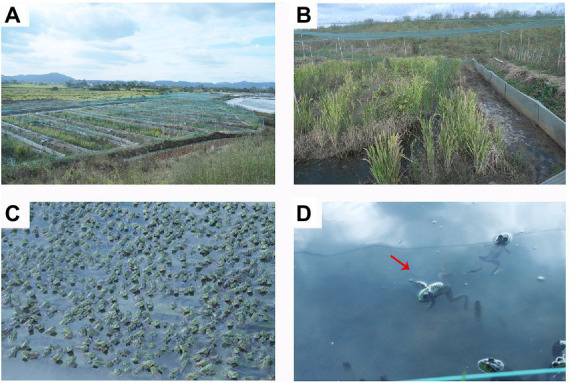
Morphology of the healthy and diseased bullfrogs (*Lithobates catesbeiana*). **(A,B)** Farm environment. **(C)** Breeding density; **(D)** Morphology of diseased (meningitis-like infectious disease, which causes muscle relaxation in sick frogs) frog morphology (red arrow).

Bullfrog, *Lithobates catesbeiana*, is the most popular large edible frog found globally (Nori et al., 2011). The glands and the bile extracted are of economic value due to the nature of skin, oil, and hormones (Cathers et al., 1997), and play a key role in aquaculture, medicine, and other industries (Bury and Whelan, 1985; Silva et al., 2009). Farmers use various techniques to improve the yield during the rearing of bullfrogs. However, the water body has limited bearing capacity, and the occurrence of diseases is positively correlated with the stocking density. The continuous, large-scale, and hence high-density of bullfrog breeding caused many issues, such as a shortage of biologically healthy food, degradation of germplasm resources (Graves and Anderson, 1987), and deterioration of the breeding environment. The lack of breeding technology has complicated the aforementioned issues. The number of diseases encountered during bullfrog breeding is on the rise becoming more detrimental and resulting in frequent large-scale outbreaks seriously hindering industrial development. The pathogens of bullfrogs that are mainly responsible for diseases include bacteria, viruses, and parasites (Zhe et al., 2013). However, the strains used in aquaculture have usually been derived from wild strains (Kibenge et al., 2012). They may not have had enough time to adapt to high-density confinement in the aquaculture environment (Rodríguez-Ramilo et al., 2011) compared with terrestrial farmed animals. This chronic stress (Yada and Nakanishi, 2002) provides opportunities for the emergence of diseases caused by pathogens that may be harmless under natural conditions. Among several pathogens, *Aeromonas hydrophila* and *A. salmonicida* are considered the most common pathogens in freshwater fish, while *Vibrio anguillarum* and *V. parahaemolyticus* are the most familiar bacterial pathogens in a marine environment, causing different types of fish diseases such as ulcer disease, carp erythrodermatitis, motile *Aeromonas* septicemia, and so forth (Lightner, 1985; Martínez Cruz et al., 2012). In December 2021, a strange disease, commonly known as meningitis-like infectious disease (MID), broke out at a bullfrog farm (Figures 1A,B) in Ma’anshan City, Anhui Province. The sick frogs showed symptoms such as head tilt, loss of motor balance, opacity or hyperemia of eye lens, photophobia, and body edema, and even death (Figure 1D).

### Microbiome and health

Most studies on the gut microbiome have been conducted in mammals, especially laboratory rodents and humans (Kohl and Yahn, 2016). However, studies on the gut microbiome of other vertebrates such as amphibians are lacking (Tong et al., 2019). The composition of frog gut microbiota is affected by season, temperature, and developmental state. Therefore, it is meaningful to study the gut and oral microbes of diseased frogs. The “ecosystem services perspective” treats the microbiota as an ecosystem that provides “services” to humans. Like any ecosystem, the host has some control over the structure of this system and the functional benefits it provides (Shreiner et al., 2015; Foster et al., 2017). The ecosystem services perspective treats the microbiome as an independent ecosystem that provides its host with services required for life and individual well-being (Calow, 1995; Arumugam et al., 2011). Gut microbial communities profoundly influence vertebrate physiology, impacting animal development, nutrition, immune function, and behavior. The gut microbiota are microorganisms (mainly bacteria but also viruses, protozoa, and fungi) and their collective genetic material present in the gastrointestinal tract (Falony et al., 2016). Besides the direct actions on the gut mucosa and enteric nervous system, many chemical mediators produced by the gut microbiome enter the bloodstream and communicate with distal organs such as the brain, heart, and liver (Evans et al., 2013), The symbiotic relationship between microbiota and the host is mutually beneficial. The host provides an important habitat and nutrients for the microbiome, and the gut microbiota support the development of the metabolic system and the maturation of the intestinal immune system by providing beneficial nutrients (D’Argenio and Salvatore, 2015), for example, by the synthesis of vitamins (Kau et al., 2011; Kitamoto et al., 2020) and short-chain fatty acids (Topping and Clifton, 2001; McDermott and Huffnagle, 2014). The oral cavity is a primary gateway to the human body and has the second-largest and most diverse microbiota after the gut, harboring > 770 species of bacteria (Kitamoto et al., 2020). A variety of microbial habitats in the oral cavity (e.g., teeth, buccal mucosa, soft and hard palate, and tongue) makes the ecologic system complex and attracts diverse microorganisms, called the oral microbiome, including bacteria, fungi, and viruses (Kilian, 2018). More than half of microbial species (e.g., *Streptococcus* and *Veillonella*) frequently detected in both sites showed evidence of oral–gut translocation, even in healthy individuals (Schmidt et al., 2019). Accumulating evidence indicates that resident oral bacteria can translocate to the gastrointestinal tract through hematogenous and enteral routes. The dissemination of oral microbes to the gut may exacerbate various gastrointestinal diseases, including irritable bowel syndrome, inflammatory bowel disease, and colorectal cancer (Kitamoto et al., 2020).

### Meningitis-like infectious disease characteristics and experimental purpose

Meningitis-like infectious disease is a highly contagious and lethal disease for bullfrogs. Once infected, it brings great losses to farmers. In the farmed frogs, the characteristics of MID were anorexia, slow movements, corneal opacity, and a series of neurological symptoms including the onset of torticollis, indifference to stimuli, intermittent motion, and curling up of toes of the limbs. The diseased frogs in this study were lethargic, and most died within a few days. Dissection revealed lesions in multiple internal organs. Histopathological lesions in the liver, spleen, kidney, heart, brain, and muscle of the diseased frogs showed cell degeneration and necrosis (Hu et al., 2017). In these studies about MID, the main pathogens were basically determined to be bacteria of the genus *Elizabethkingia* (Lei et al., 2019). Relevant cases have been reported in bullfrogs (Mauel et al., 2002), Chinese spiny frogs (*Quasipaa spinosa*; Lei et al., 2019), black-spotted frogs (Hu et al., 2017; Chang et al., 2021), tiger frogs (Xie et al., 2009), adult northern leopard frogs (*Lithobates pipiens*), chapa bug-eyed frogs (*Theloderma bicolor*), Vietnamese warty toads (*Bombina microdeladigitora*), and Sabana Surinam toads (*Pipa parva*; Trimpert et al., 2021). This experiment started with gut and oral microbes to examine the differences in the gut and oral microbes between normal and diseased individuals so as to explore the causes of disease outbreaks in bullfrogs raised in Dayutan. The pathogenic-related bacterial communities were revealed through the differences between the biological groups, providing a theoretical basis for the prevention and control of MID in the later stage.

## Materials and methods

### Oral and gut samples collection

In the study, a total of 10 bullfrogs from Dayutan in Anhui Province in 2021 were prospectively collected. Samples are generally normal, half are sick, and the samples were selected according to the criteria: (1) All samples are from the same culture pond; (2) about the similar size; (3) age < 1 year old; (4) Breeding conditions were the same before collection; The bullfrog was dissected in a sterile environment, and its gut and oral microbiome samples were taken, 10 cases (gut and oral) of sick group (MID), 10 cases (gut and oral) of healthy control group (HC) were finally included in the study and received 16S rDNA amplicon sequencing.

### DNA extraction and PCR amplification

Ten bullfrogs were dissected in a sterile environment, the intestines and orals were carefully scraped with a sterile swab, the swabs after sampling were marked, and the water samples were drawn with a 0.22 μm filter membrane in a sterile environment. After filtration, placed the filter membrane in a sterile Ep tube for marking. The genomic DNA of the sample was extracted by CTAB method (Miaomiao et al., 2008), and then the purity and concentration of the extracted DNA were detected by agarose gel electrophoresis.

### Library construction and sequencing

PCR amplification of 16S rRNA gene sequences was performed by targeting the V3–V4 regions by using a set of forward and reverse primers according to a previous study (Chen et al., 2022). The sequences of forward and reverse primers used in this experiment were as follows: Pro_341F (5′-ACTCCTACGGGAGGCAGCA-3′) and Pro_806R (5′-GGACTACHVGGGTWTCTAAT- 3′; Nossa et al., 2010). The PCR was carried out in a total volume of 25 μl that contains 2 × KAPA HiFi HotStart Ready Mix (12.5 μl), 400 nM of each primer, ddH_2_O (7.5 μl) and 3 μl of template DNA. The PCR reaction was carried out in a thermocycler (Px2 Thermal Cycler, Thermo, United States) under the following conditions: initial denaturation at 95°C for 30 s followed by 28 cycles of denaturation at 95°C for 30 s, annealing at 55°C for 30 s and elongation at 72°C for 30 s. The amplicons were confirmed by electrophoresis with 1.5% agarose gel under 110 V for 30 min. After separation using agarose gel electrophoresis, PCR products with expected sizes were purified from the matrix. The amplicons were subjected to sequencing using the pair-end method with the MiSeq Illumina platform (Illumina Inc., San Diego, CA, United States; Quail et al., 2008), following manufacturer instructions. The Nextera XT DNA Library Preparation Kit (Illumina) was used to construct libraries from the isolated DNA.

### Operational taxonomic unit (OUT) clustering and species annotation

Using Cutadapt (V1.9.1) cut the low quality part of reads; the sample data were separated from the obtained reads according to Barcode, and the Barcode and primer sequences were truncated to obtain raw reads. The Reads sequence was compared with the species annotation database, the chimera sequence was detected and removed, and the Clean Reads were obtained. All the Clean Reads of all samples were clustered by Uparse software (Uparse v7.0.1001; Edgar, 2013). The sequences were clustered into Operational Taxonomic Units (OTUs), with 97% identity and the sequences with the highest frequency were taken as the representative sequences of OTUs according to the principle of algorithm. Then the species annotation of OTUs sequence was analyzed by Mothur method (Schloss et al., 2009) and SILVA132 SSUrRNA database (Set the threshold to 0.8–1; Apprill et al., 2015), and the taxonomic information was obtained.

### Statistical analysis

The differences between groups were compared by calculating the α index (Observed-species, Chao1, Shannon, Simpson) of different groups with *t*-test. The relative abundance at the genus level was calculated. A Bray–Curtis dissimilarity matrix was calculated based on relative abundance of OTUs and used to perform non-metric multidimensional scaling (NMDS). We used the linear discriminant analysis (LDA) effect size (LEfSe) to identify significant associations between bacterial taxa and different groups. LEfSe can determine the taxonomic units most likely to explain differences between classes by coupling standard tests for statistical signifcancewith additional tests encoding biological consistency and effect relevance (LDA score > 2.0, *p* < 0.05).

## Results

### Increased microbiome abundance in the mid group

**Figure 2:**
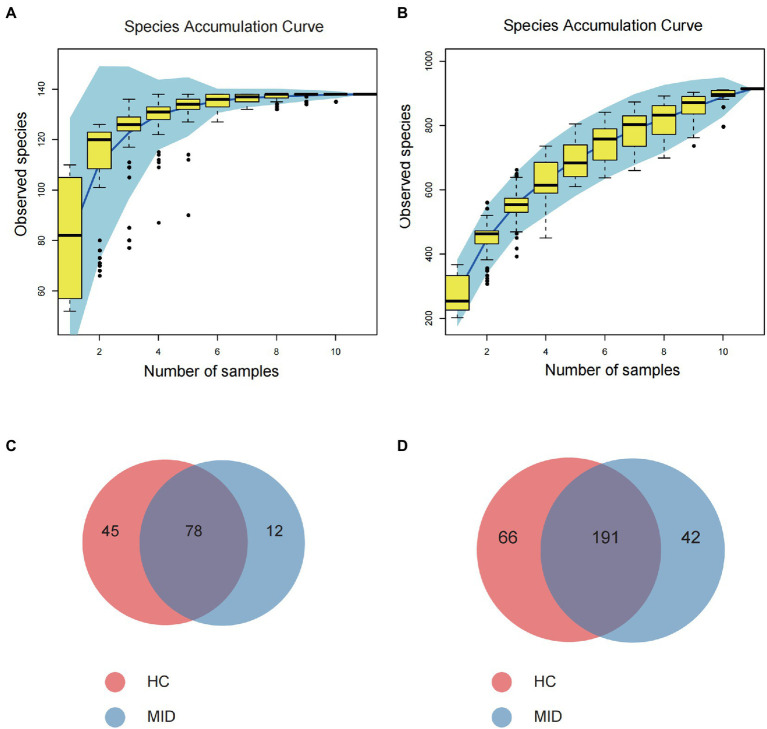
Rationality test of sample number and component difference statistics. Rarefaction Curve, horizontal axis is the number of sequencing strips randomly selected from a sample, and vertical axis is the number of OTU that can be constructed based on the number of sequencing strips, which is used to reflect the sequencing depth. The results showed that with the increase of the sample size, the number of OTU in the gut cavity **(A)** and oral cavity **(B)** gradually stabilized, indicating that the sampling number was reasonable. Venn diagram **(C,D)** showing significantly differential OTU between healthy control (HC) and meningitis-like infectious disease (MID). **(C)** Shows intestinal results and **(D)** shows oral results.

High-throughput sequencing of the 16S rDNA gene V3–V4 region was performed in 20 samples to examine the structure of the gut and oral microbiome. The basic data statistics after sequencing are shown in Tables 1, 2. The accumulation curve analysis showed that the rarefaction curve of the gut (Figure 2A) and oral cavity (Figure 2B) tended to be flat, indicating that the amount of sequencing data was gradually reasonable, and more data would only produce a small number of new species (OTUs). Therefore, the sample size of the study was sufficient, the sequencing depth was up to the standard, and an additional sample size was not required. In the gut cavity, 135 OTUs were obtained; of 78 in the two groups, 45 OTUs were unique to the MID group, and 12 were unique to the HC group (Figure 2C). Further, 299 OTUs were obtained in the oral cavity; of 191 in the two groups, 66 OTUs were unique to the MID group, and 42 were unique to the HC group (Figure 2D).

**Table 1:** Basic data for sequencing gut microbiome samples.

Group	Reads-raw	Reads-derep	Average Reads-derep
HC	149,161	121,237	97,876
	92,792	70,279	
	115,658	90,554	
	126,904	99,338	
	140,068	107,973	
MID	119,440	59,623	70,080
	96,755	53,670	
	167,344	89,733	
	99,351	57,747	
	112,488	89,625	

**Table 2:** Basic data for sequencing oral microbiome samples.

Group	Reads-raw	Reads-derep	Average Reads-derep
HC	130,389	79,633	64,549
	104,094	50,821	
	98,669	49,365	
	133,214	70,968	
	153,602	71,960	
MID	129,252	83,286	100,243
	195,408	128,809	
	179,203	100,140	
	143,606	94,194	
	145,760	94,784	

### Alterations in microbiomes in the mid group

**Figure 3:**
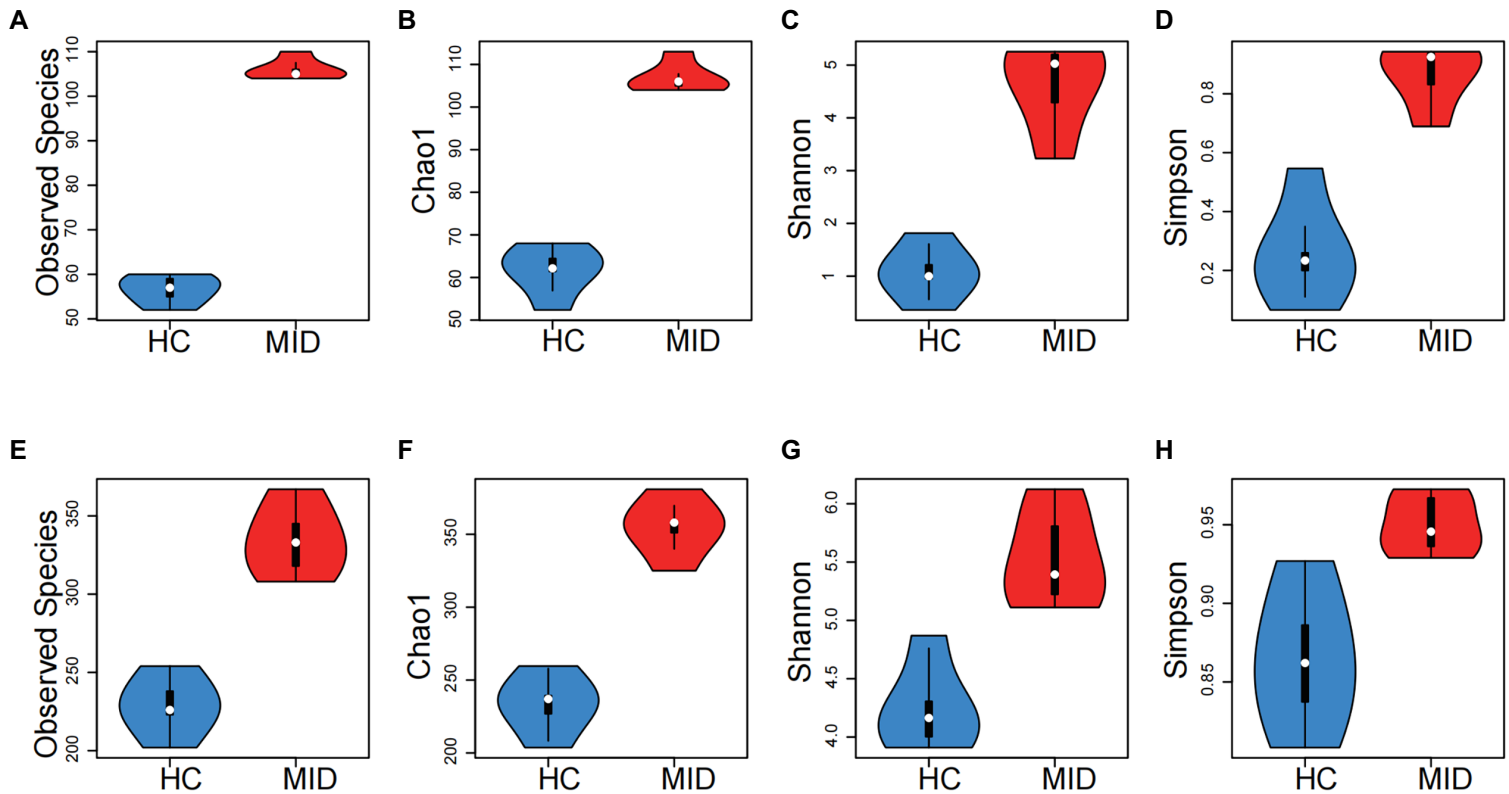
Group differences between oral **(A–D)** and intestinal **(E–H)** samples, MID group had significant higher diversity in both oral and intestinal microbiota than that of HC group (*p* < 0.05 for all tests, *t*-test). The horizontal axis is the grouping, and the vertical axis is the alpha indexes (Observed specifications, Chao1, Shannon and Simpson). The white point represents the mean value, the outer contour represents the sample distribution, the upper side of the black box in the middle represents the upper 1/4, and the lower side represents the lower 1/4.

In the oral cavity, the *α* indexes, observed species (*p* = 6.014 × 10^−5^), Chao1 (*p* = 1.237 × 10^−5^), Shannon (*p* = 0.001), and Simpson (*p* = 0.0099; Figures 3A–D), had significant differences. No significant difference was observed in the abundance and evolutionary distance of microorganisms between the HC and MID groups, but a significant difference was observed in the richness and evenness. In the gut cavity, the α indexes were significantly different between the HC and MID groups, including observed species (*p* = 1.079 × 10^−8^), Chao1 (*p* = 2.439 × 10^−6^), Shannon (*p* = 0.00013), and Simpson (*p* = 0.0004; Figures 3E–H). Except for Simpson and Shannon, the *α* indexes had very significant differences. This indicated a large difference in the richness and evenness of microorganisms in the gut cavity between the HC and MID groups.

### Bacterial taxonomic abundance at the phylum and genus levels based on 16S rRNA gene amplicons

**Figure 4:**
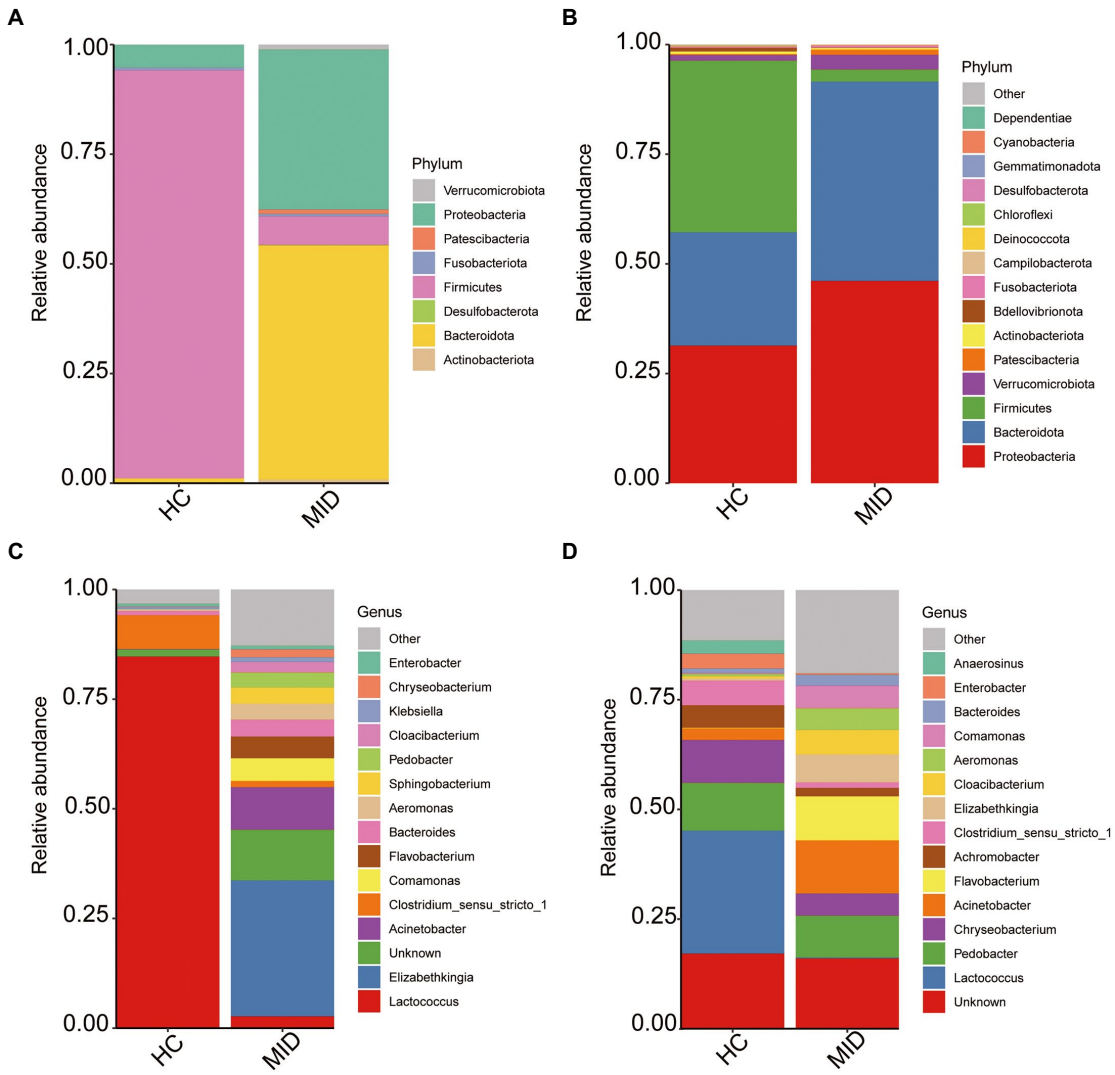
Average relative abundance of phylum and gene levels. The top 15 phylum **(A,B)**, and genus **(C,D)**, with the highest abundance in each group were presented and the rest were set to others.

The top 15 bacterial phyla in the HC and MID groups were evaluated by examining the changes in the gut and oral microbiota. The results showed that *Proteobacteria* was the most abundant phylum in the oral cavity in the HC group (Figure 4B), followed by *Bacteroidota* and *Firmicutes*. However, the *Firmicutes* abundance decreased significantly and the *Bacteroidota* abundance increased significantly in the MID group compared with the HC group. Only eight phyla of microorganisms were found in the gut (Figure 4A). The *Firmicutes* abundance significantly decreased and the *Bacteroidota* abundance significantly increased in the MID group, which was consistent with those in the oral cavity. At the genus level, *Lactococcus* was the dominant bacteria in the gut in the HC group (Figure 4C). However, almost no *Lactococcus* was found in the MID group, while the relative abundance of *Flavobacterium* and *Acinetobacter* increased significantly. The relative abundance of *Lactococcus* decreased sharply and the relative abundance of *Flavobacterium* and *Acinetobacter* increased significantly in the MID group, which was consistent with the abundance in the oral cavity (Figure 4D). Different from the oral manifestations, *Flavobacterium* and *Acinetobacter* were the dominant bacteria in the intestinal tract in the MID group.

**Figure 5:**
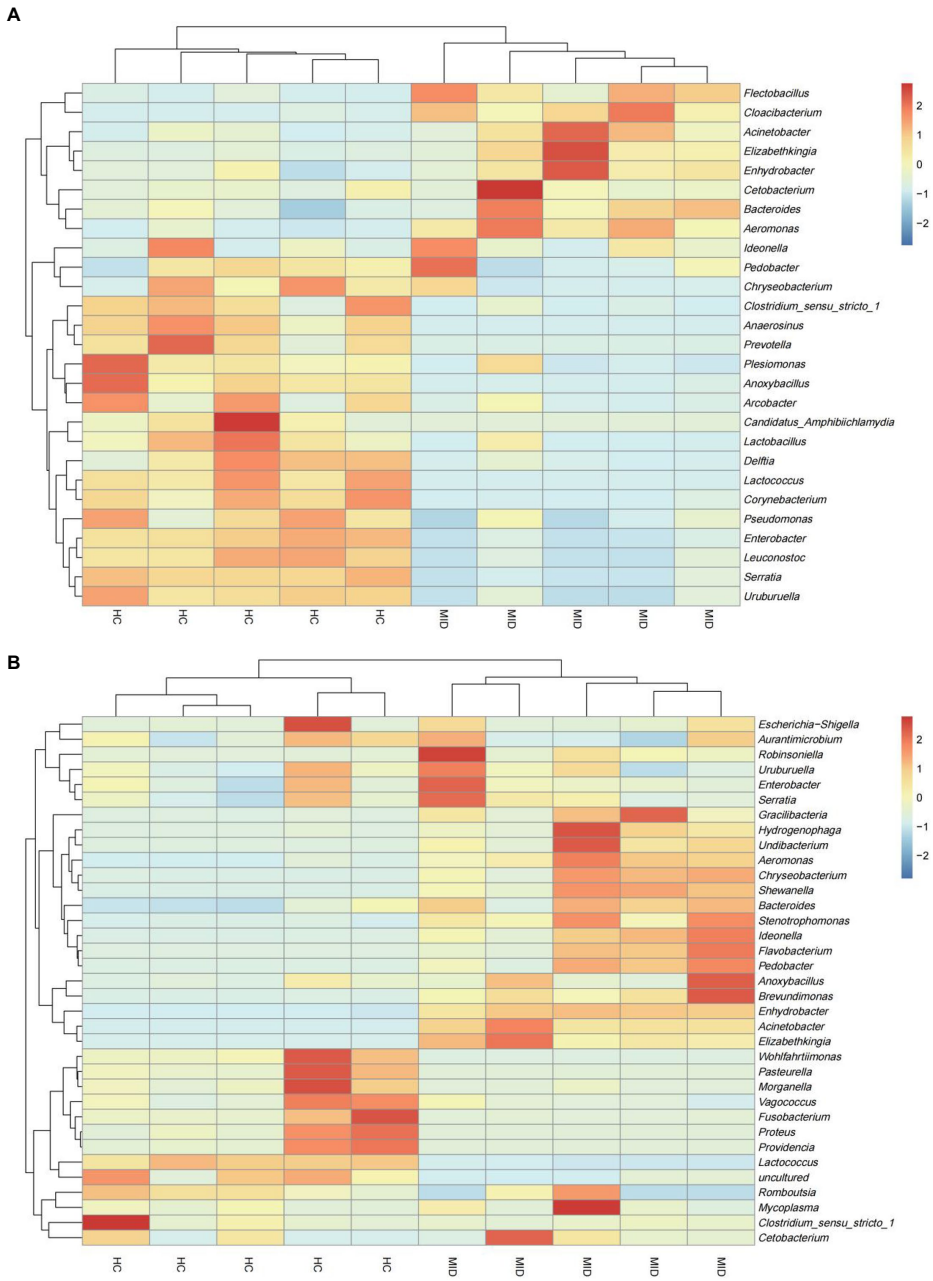
Heat map of the genus of top 35 microbiota in the HC and MID groups. Differences in relative abundance at genus level in different samples. Heat map showed the difference in annotated gene abundance at genus level among groups (showing the relative abundance of the top 35 genus). The row clustering distance method is Pearson correlation, and the default is Euclidean distance. Columns represent a sample and rows represent a genus. Based on the average relative abundance of the genus in the same sample, the expression level higher than the average value is positive and marked in red; on the contrary, the expression level below the average value is negative and marked in blue. The shade of color indicates the degree of difference between the relative abundance and the mean. The dendrogram above the main body of the heatmap clusters the source of the samples, which is convenient for distinguishing different samples (normal and diseased); the dendrogram on the left of the heatmap clusters the relative abundance of genera, and groups the genera with similar relative abundance to For one class, the color difference is more pronounced. In the gut **(A)**, genera with high relative abundance were concentrated in the diseased group, and in the oral cavity **(B)**, genera with high relative abundance were concentrated in the normal group.

We considered the relative abundances of the top 35 genera of the samples to determine the microbiota of high abundance. The microbiota of high abundance analysis revealed that *Gracilibacteria*, *Hydrogenophaga*, *Undibacterium*, *Aeromonas*, *Chryseobacterium*, *Shewanella*, *Bacteroides*, *Stenotrophomonas*, *Ideonella*, *Flavobacterium*, *Pedobacter*, *Anoxybacillus*, *Brevundimonas*, *Enhydrobacter*, *Acinetobacter*, and *Elizabethkingia* were the microbiota of high abundance in the gut in the MID group (Figure 5A). The microbiota of high abundance were *Flectobacillus*, *Cloacibacterium*, *Acinetobacter*, *Elizabethkingia*, *Enhydrobacter*, *Cetobacterium*, *Bacteroides*, and *Aeromonas* in the oral cavity (Figure 5B) in the MID group. *Elizabethkingia*, *Enhydrobacter*, *Bacteroides*, and *Aeromonas* were found in the oral cavity and gut. It was observed that the oral flora structure had changed during the disease process, and excessive reproduction of some pathogenic bacteria had inhibited the structure of the original healthy flora. The structure of the intestinal flora also changed in the gut tract due to the excessive reproduction of pathogenic bacteria. Further, the abundance of pathogenic bacteria overexpressed in the intestinal tract was much higher than that in the oral cavity in the HC group.

### Differences in the microbial community structure between HC and mid groups

Non-metric multidimensional scaling (NMDS) showed the structural differences in the microbiota based on two groups. Bray–Curtis showed that gut (Figure 5A) and oral (Figure 5B) microbiota were differentially distributed in the MID and HC groups. Hence, the microbiota of both oral cavity and gut segments could be divided into two distinct groups, and the two groups displayed apparent differences (stress = 0.0012 and 0.0131, respectively).

**Figure 6:**
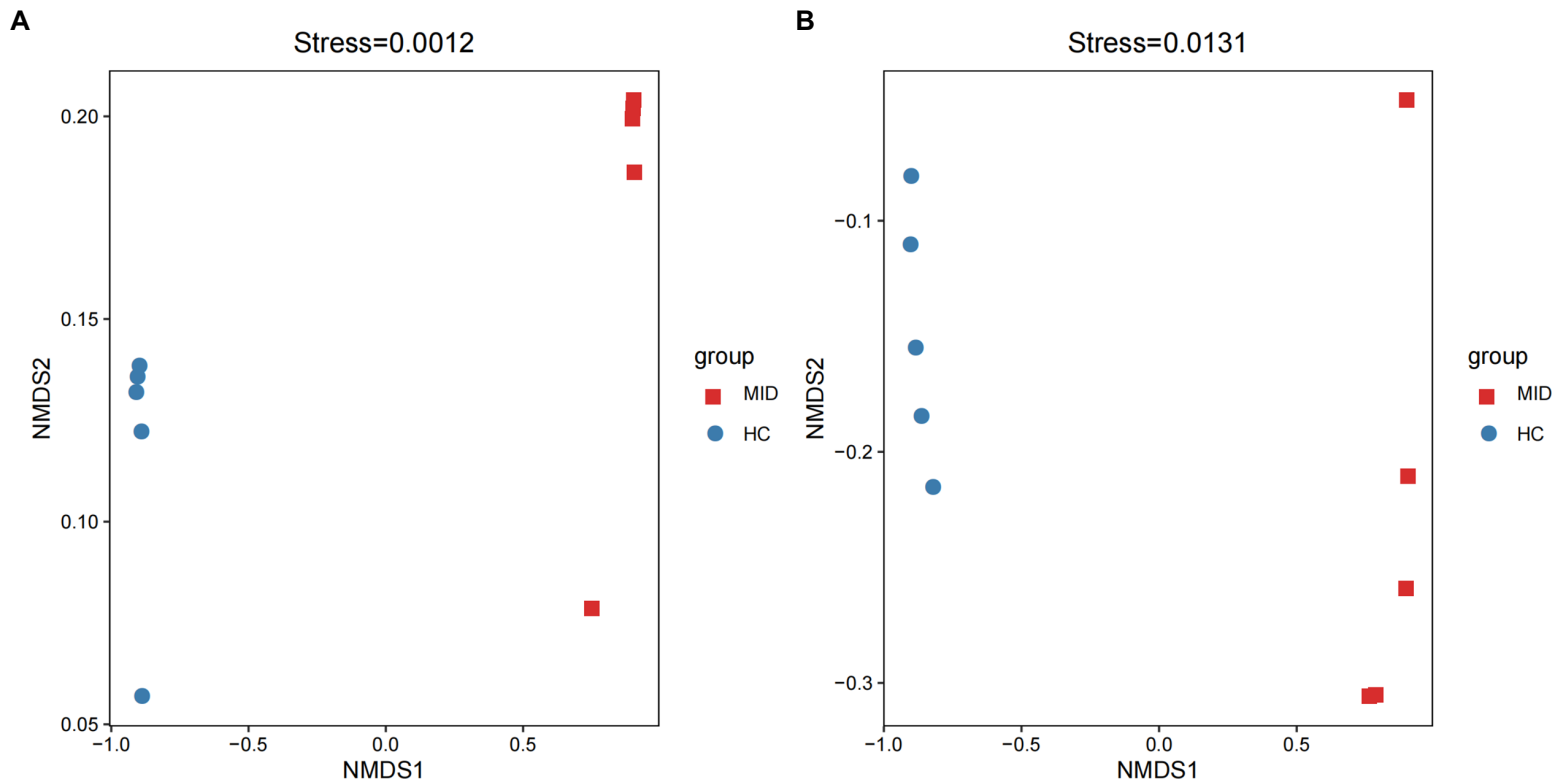
Non-metric multidimensional scaling (NMDS) of the microbial communities of different groups. Both gut **(A)** and oral **(B)**, all samples can be divided into two categories according to MID and HC.

**Figure 7:**
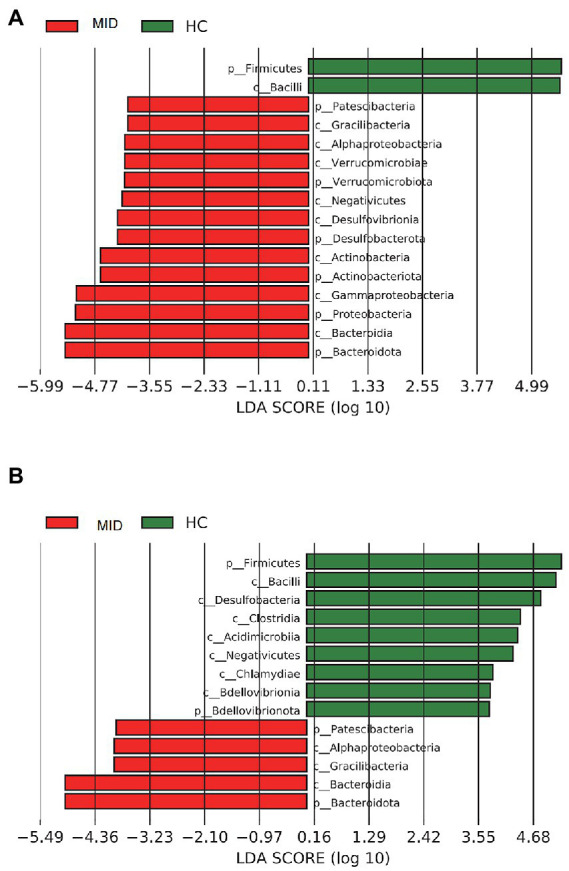
Bacterial of different levels in MID and HC. Samples from MID and HC were compared using LEfSe; the bars depict bacterial with significantly different relative abundance. Bars represent linear discriminant analysis (LDA) scores. **A** is from a gut sample and **B** is from an oral sample.

In the gut, the LEfSe analysis showed that 16 microbial clades exhibited significant differences between the MID and HC groups (Figure 6A). In the HC group, *Firmicutes* and *Bacilli* were highly abundant. In the MID group, *Bacteroidota* had the highest LDA score. In the oral cavity, the LEfSe analysis showed that 14 microbial clades exhibited significant differences between the MID and HC groups (Figure 6B). In the HC group, *Firmicutes* and *Bacilli* were highly abundant. In the MID group, *Bacteroidota* had the highest LDA score (Figure 7).

## Discussion

In this study, 16S amplicon sequencing was used to detect the differences in gut and oral microbiota composition and population numbers of diseased and normal bullfrogs to find the pathogenic bacteria that caused the disease in bullfrogs.

### Differences in microbiome structure

In humans, there are many reports of disease and gut microbes. For example, chronic kidney disease (CKD) will reduce the richness, uniformity and uniformity of intestinal microorganisms (Loomba et al., 2017); Non-alcoholic fatty liver disease (NAFLD) will reduce the diversity of intestinal microorganisms α and β (Astbury et al., 2020). But we calculated the relative abundance of oral and gut microorganisms in normal and diseased frogs and we were found that the diversity, uniformity and richness of the flora in MID increased. It might be due to the decline in the immune function of the body declined after the pathogenic bacteria infected the body resulting in some conditional pathogenic bacteria in the water body, further infecting the body. Thus, the richness of the microbial community increased.

The 16S rRNA gene amplicon sequencing could be used to determine the genetic composition and community function of all microorganisms in environmental samples (Goldfeder et al., 2017). In our study, the results of 16S rRNA gene amplicon sequencing showed that the Chao1 indexes slightly increased in the HC group compared with the MID group, but with no significant difference. Previous studies showed that a balanced gut microbial structure was an important guarantee for the health of an organism. Once the gut microbiota is unbalanced, it causes a variety of diseases; the beneficial bacteria in the gut are the key to maintain the balance of the gut microbial population structure (Qi et al., 2019). In the HC group, the abundance of *Bacteroidetes* and F*irmicutes* accounted for 97% of the total bacteria*. Lactococcus* has been used in dairy fermentation for centuries. These Gram-positive, generally nonpathogenic, nonmotile, and nonsporulating bacteria are members of the *Streptococcaceae* family, which includes food, commensal, and virulent species. Many probiotics in this genus are beneficial to the survival of the host. Most widely used probiotic bacteria belong to the *Lactobacillus* and *Bifidobacteria* genera, but other microorganisms, such as *Lactococcus* and *Enterococcus*, are also used as the components of probiotic preparations (Kim et al., 2019).

### *Elizabethkingia*—potential pathogenic bacteria

Currently, the reports only clarified that *Elizabethkingia* was a pathogenic bacteria, but did not explain the structural and functional changes in microorganisms after infection. In this study, the relative abundance of *Elizabethkingia* was significantly higher in the MID group than in the HC group. Our study showed that the abundance of *Elizabethkingia* increased significantly in the MID group compared with the HC group. Many studies showed various pathogenic bacteria of the genus *Elizabethkingia*, which could cause diseases in amphibians, reptiles, and even humans. *Elizabethkingia meningoseptica* was detected in tiger frogs with cataracts (Monteagudo-Mera et al., 2011), suggesting that it could cause disease in tiger frogs. Its symptoms are the same as those of the sick frogs in this study. *E. meningoseptica* has been reported to cause severe sepsis in humans (Xie et al., 2009). Cases of *E. meningoseptica* causing patient shock have also been reported, suggesting that species in this genus cause disease in humans. The genus *Elizabethkingia* has six species, two of which have been reported to be pathogenic to organisms; meningitis-like symptoms occur in both cases. Amphibians and mammals are infected by the bacteria, but no infection in reptiles has been reported. It is believed that the bacteria may also infect species similar to crocodiles and other aquatic reptiles, which will be the focus of future investigations. However, in this study, the lack of basic information such as body weight and blood routine led to the inability to analyze the differences in physiological indicators between MID and HC. In future studies, we should pay attention to recording basic data to make the research content more complete.

## Conclusion

The structure of the oral and intestinal flora of diseased frogs changed significantly. Also, the *Elizabethkingia* abundance increased remarkably, implying that the presence of *Elizabethkingia* might cause diseases in frogs. Further, the proliferation of other bacteria after the illness also further exacerbated the deterioration of the disease. It showed a significant increase in the diversity of microbiomes in both the oral cavity and the intestinal tract after the illness. It might be due to the decline in the immune function of the body after the pathogenic bacterial infection, leading to some conditional pathogenic bacteria in the water body further infecting the body and resulting in an increase in the richness of the microbial community.

## Data availability statement

The original contributions presented in the study are publicly available. This data can be found here: NCBI, PRJNA896736.

## Author contributions

TP led the research team. TP, LS, and WL designed the research. WL, GF, KS, EL, and TP collected samples. WL, GF, and TP performed research. WL, JRL, JYL, YW, and KS analyzed data. WL, GF, and KS wrote the paper. All authors contributed to the article and approved the submitted version.

## Funding

This work was supported by National Natural Science Foundation of China (No. 31872253), Anhui Natural Science Foundation (Youth, 1908085QC127), Outstanding Innovative Research Team for Molecular Enzymology and Detection in Anhui Provincial Universities (2022AH010012), Research start-up funds of Anhui Normal University (No. 751865), Province and Student Innovation and Entrepreneurship Training Program, Anhui Forestry Science and Technology Innovation Project (AHLYCX-2021-01).

## Conflict of interest

The authors declare that the research was conducted in the absence of any commercial or financial relationships that could be construed as a potential conflict of interest.

## Publisher’s note

All claims expressed in this article are solely those of the authors and do not necessarily represent those of their affiliated organizations, or those of the publisher, the editors and the reviewers. Any product that may be evaluated in this article, or claim that may be made by its manufacturer, is not guaranteed or endorsed by the publisher.
