# Supplementary figures and images for: Microbial community structure dynamics of invasive bullfrog with meningitis-like infectious disease
Source: Front Microbiol. 2023 Mar 13;14:1126195. doi: 10.3389/fmicb.2023.1126195 (PMC10040567; doi:10.3389/fmicb.2023.1126195)

**A**

Stress=0.0012

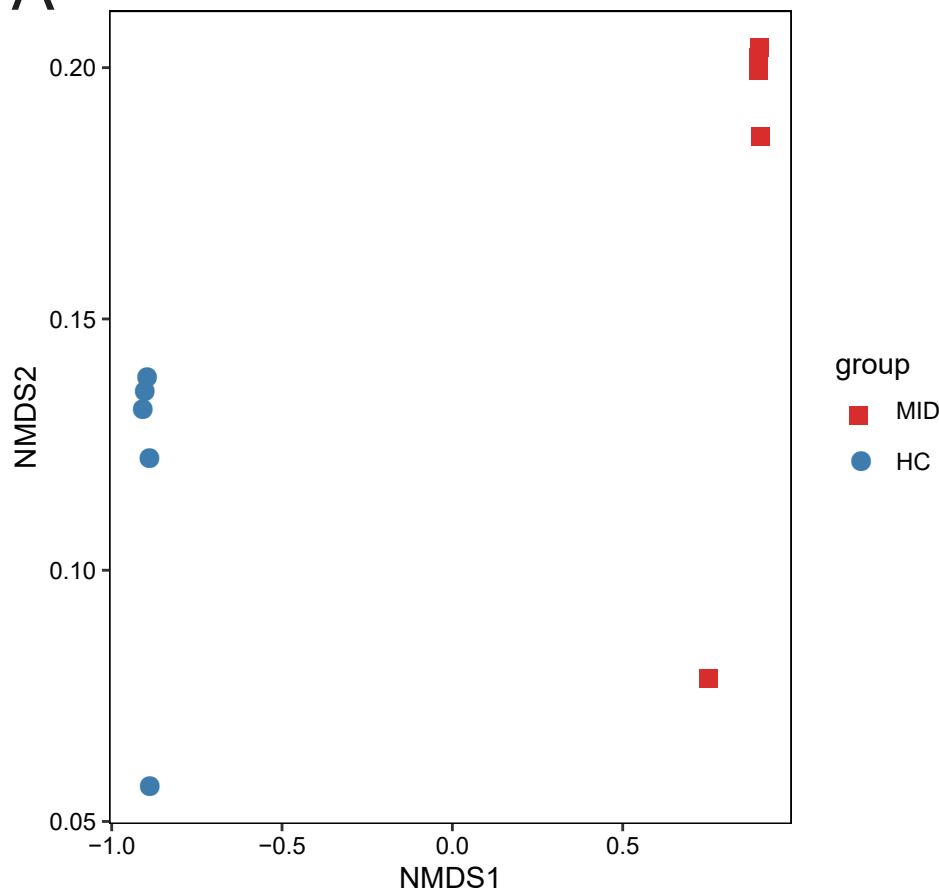**B**

Stress=0.0131

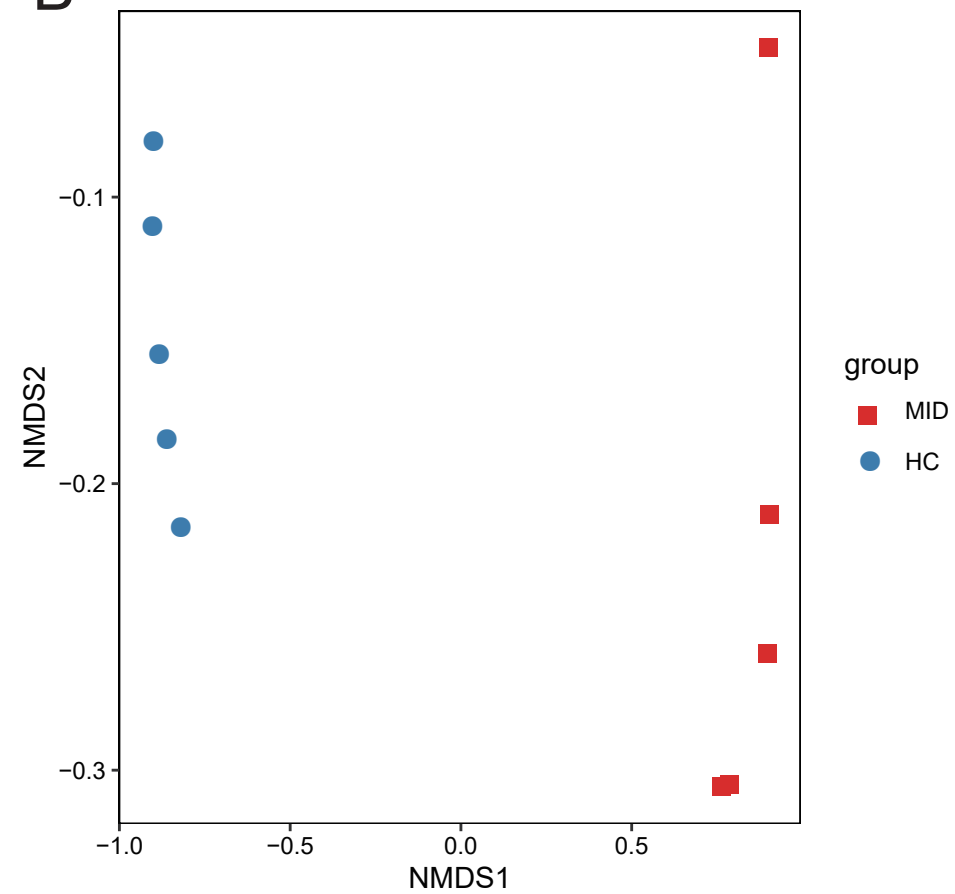

Supplement: Supplementary file 1 [file Data_Sheet_1.pdf]

A

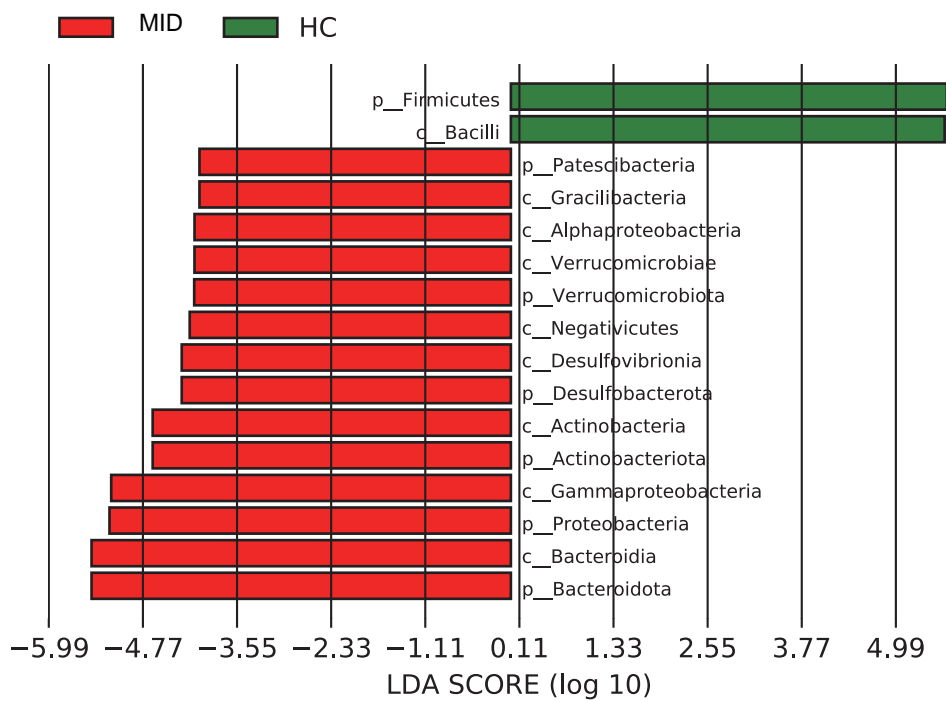

B

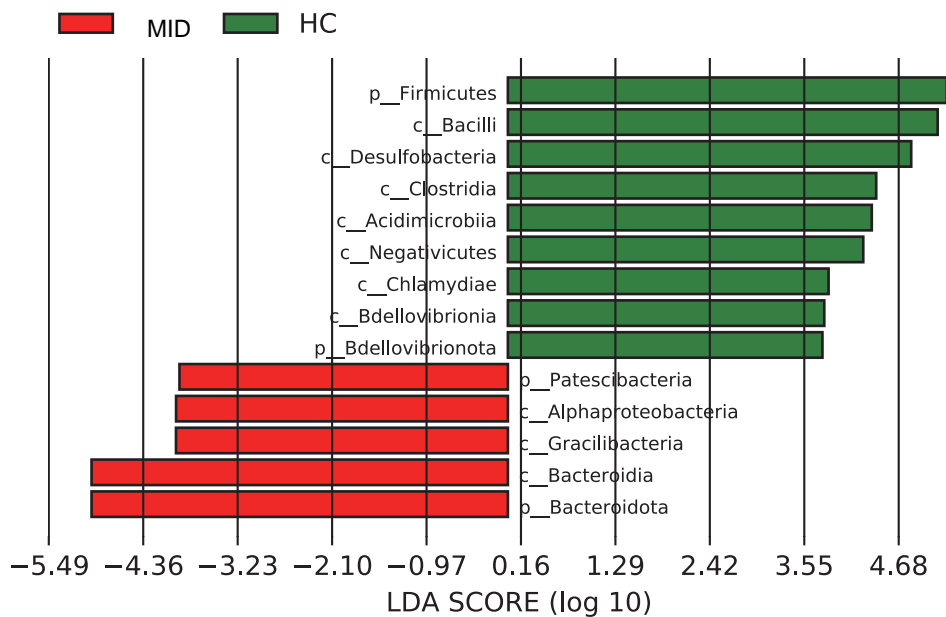

Supplement: Supplementary file 2 [file Data_Sheet_2.pdf]

**A**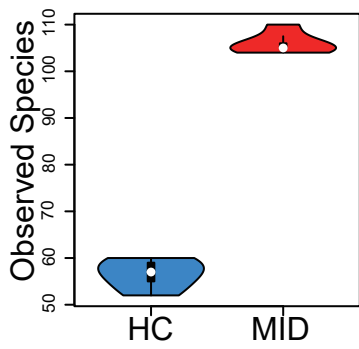**B**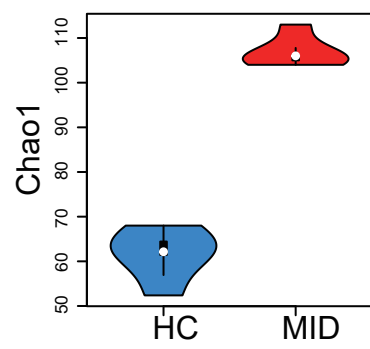**C**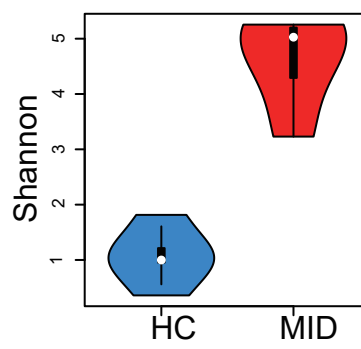**D**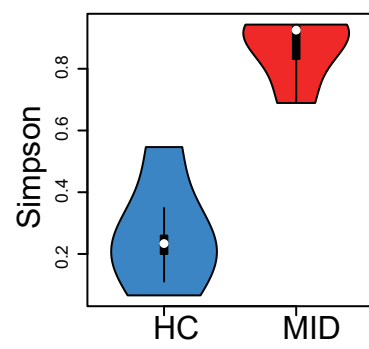**E**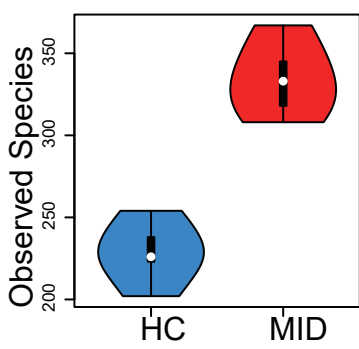**F**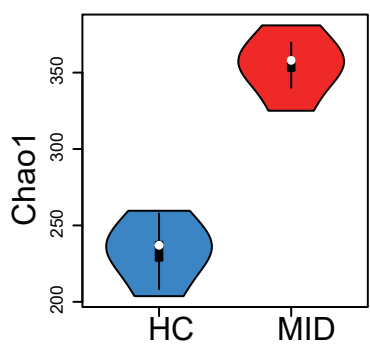**G**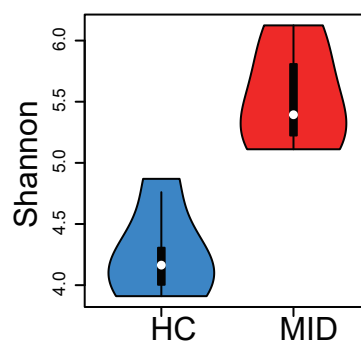**H**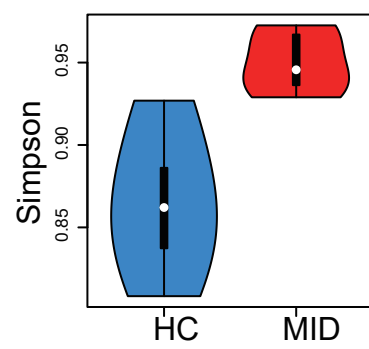

Supplement: Supplementary file 3 [file Data_Sheet_3.pdf]

A

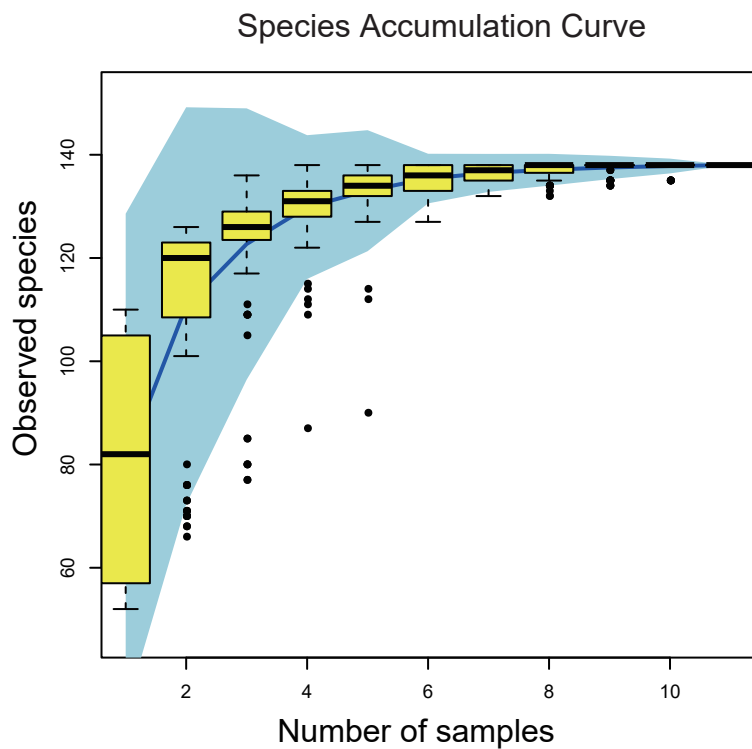

B

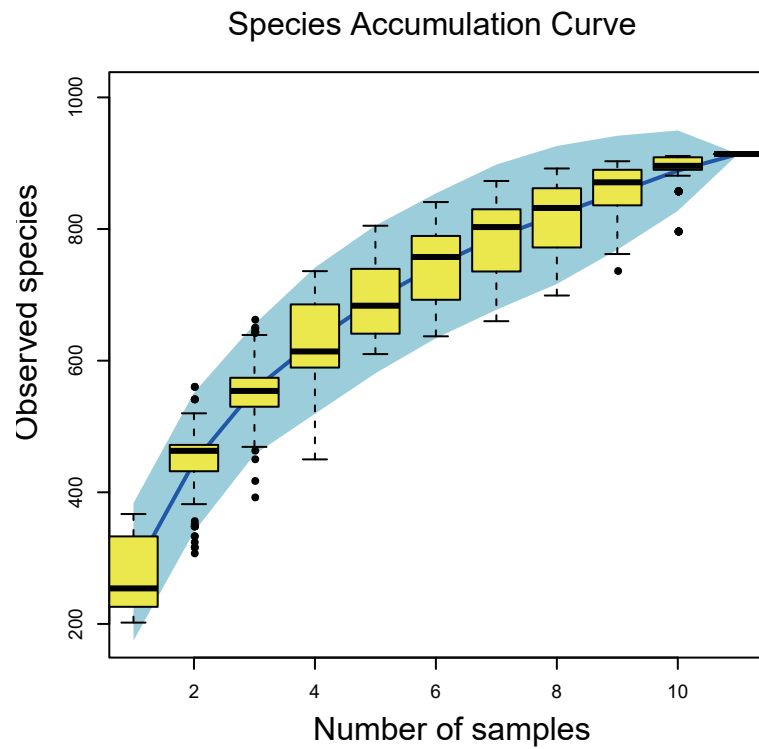

C

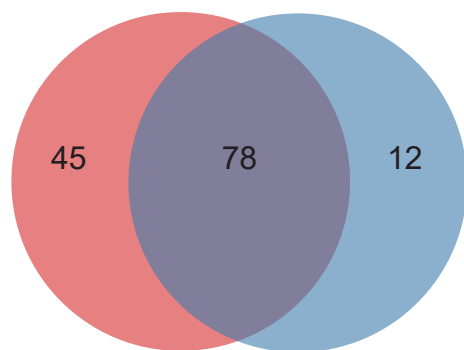

HC

MID

D

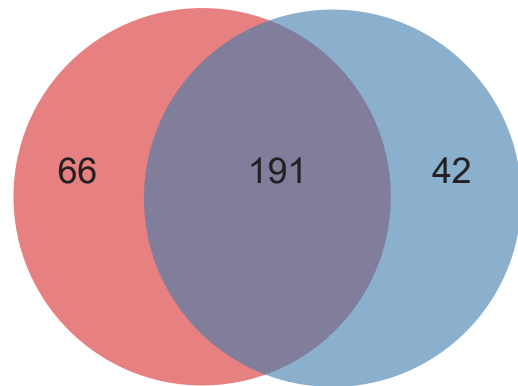

HC

MID

Supplement: Supplementary file 4 [file Data_Sheet_4.pdf]

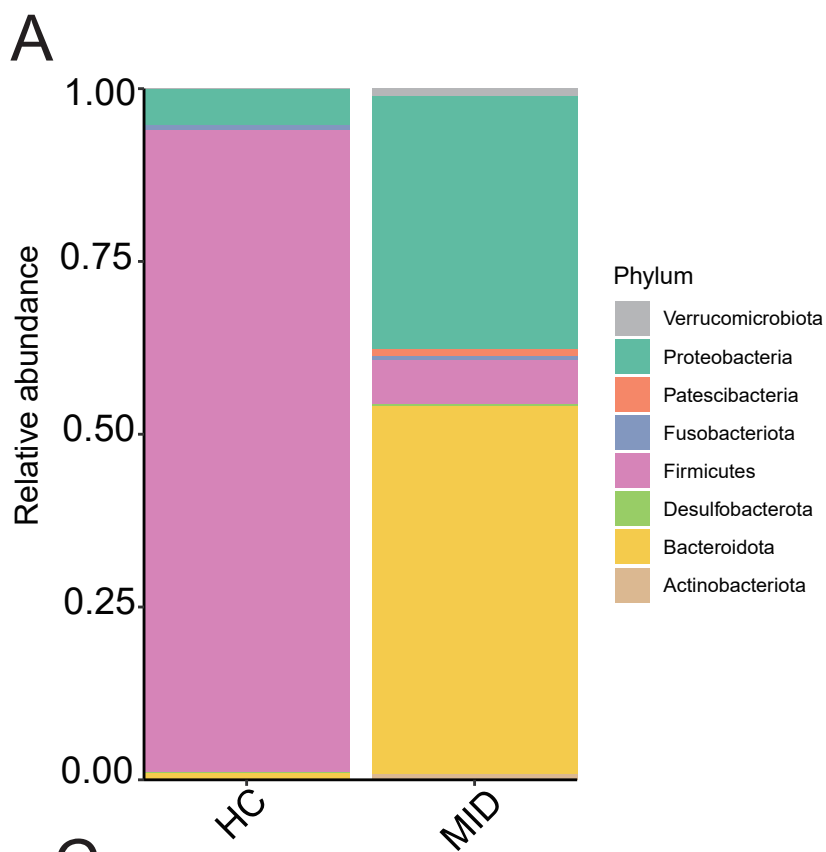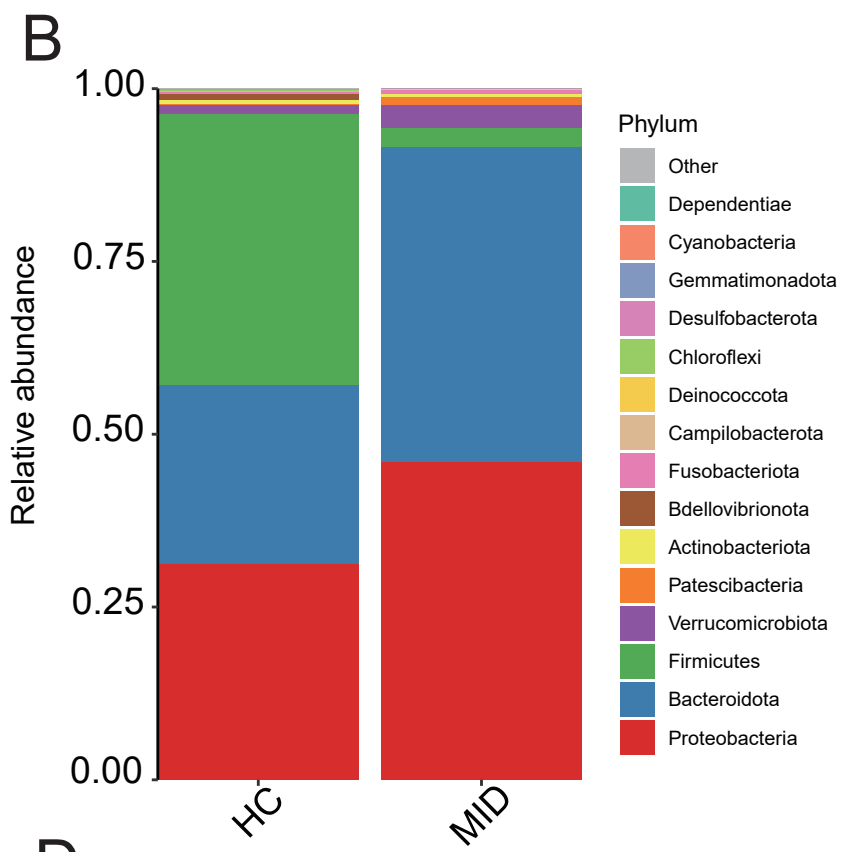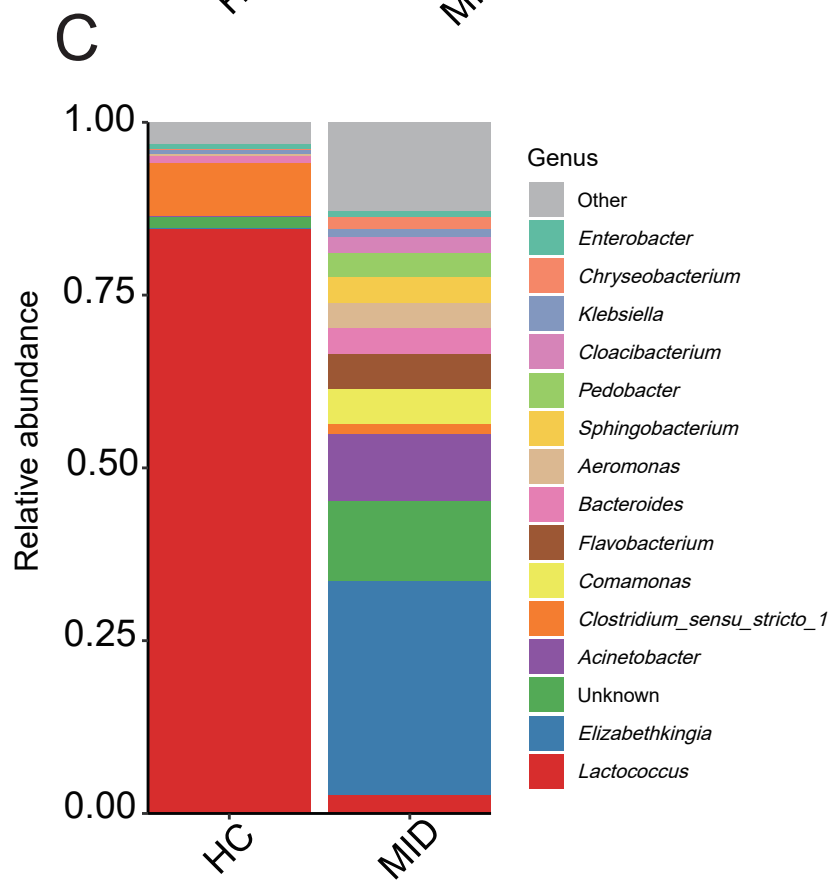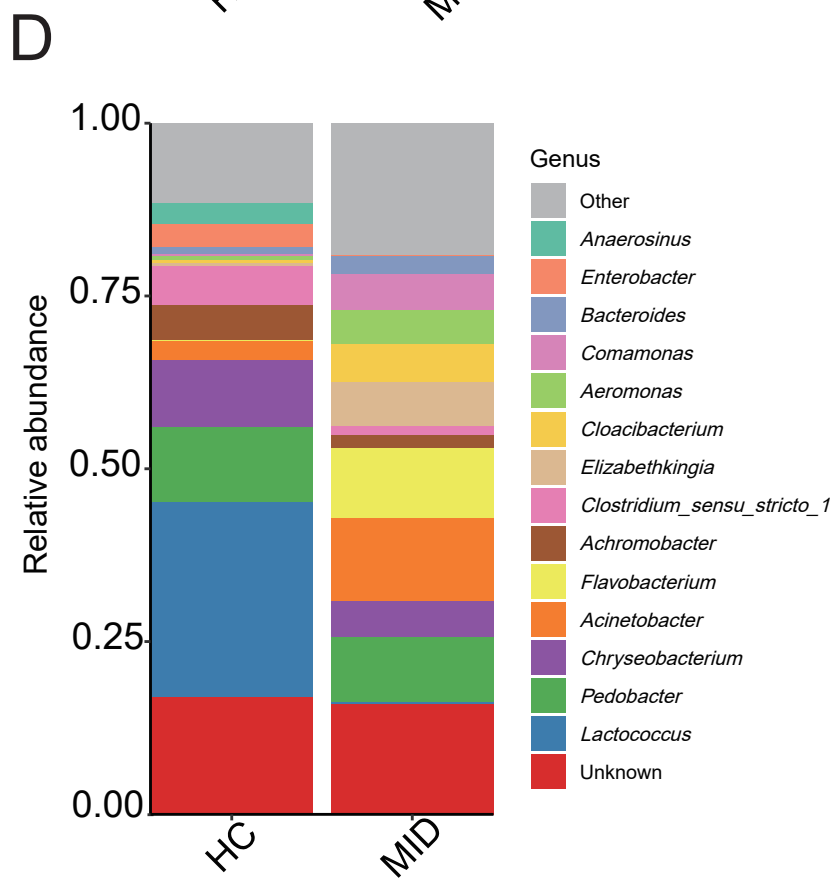

Supplement: Supplementary file 5 [file Data_Sheet_5.pdf]

A

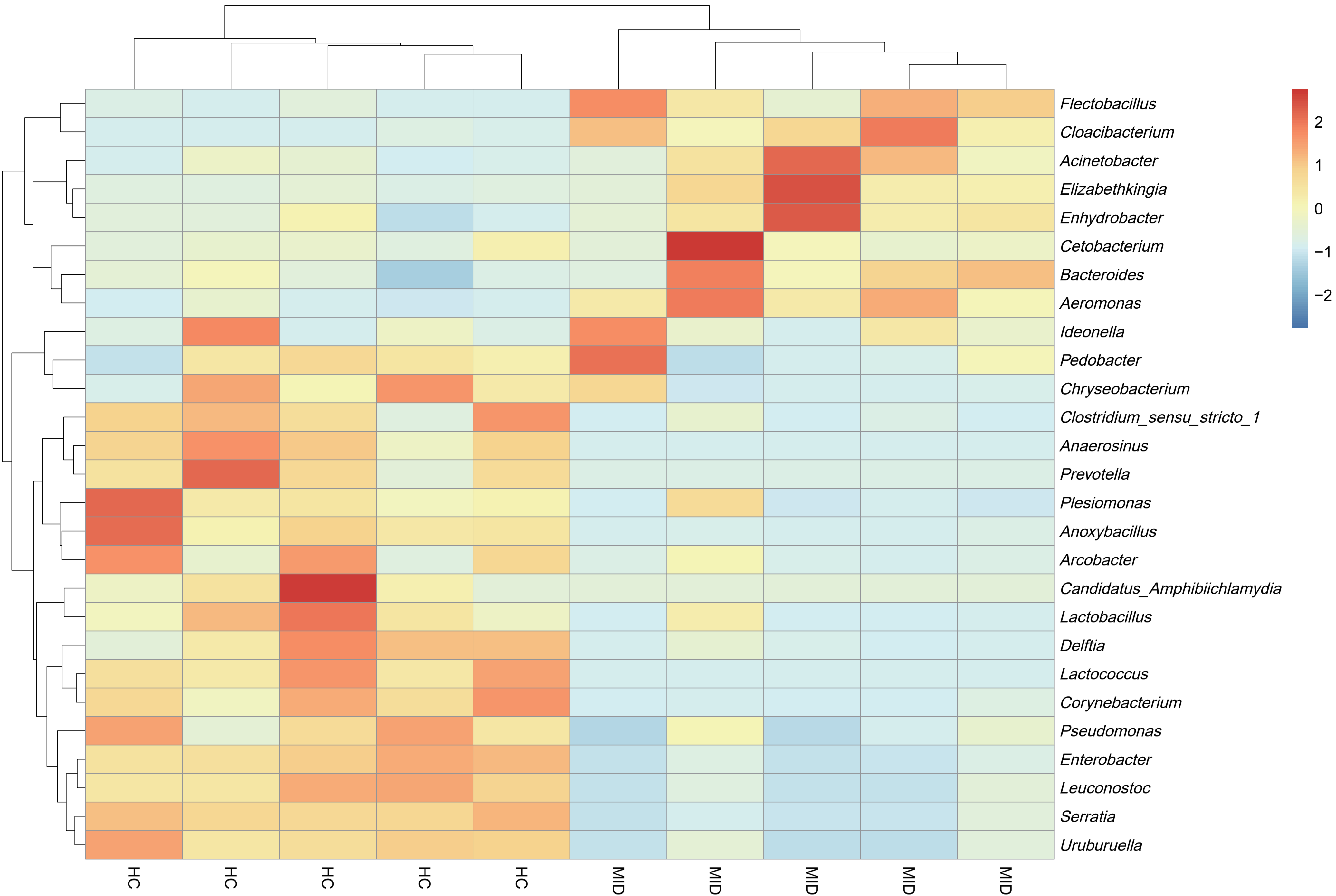

B

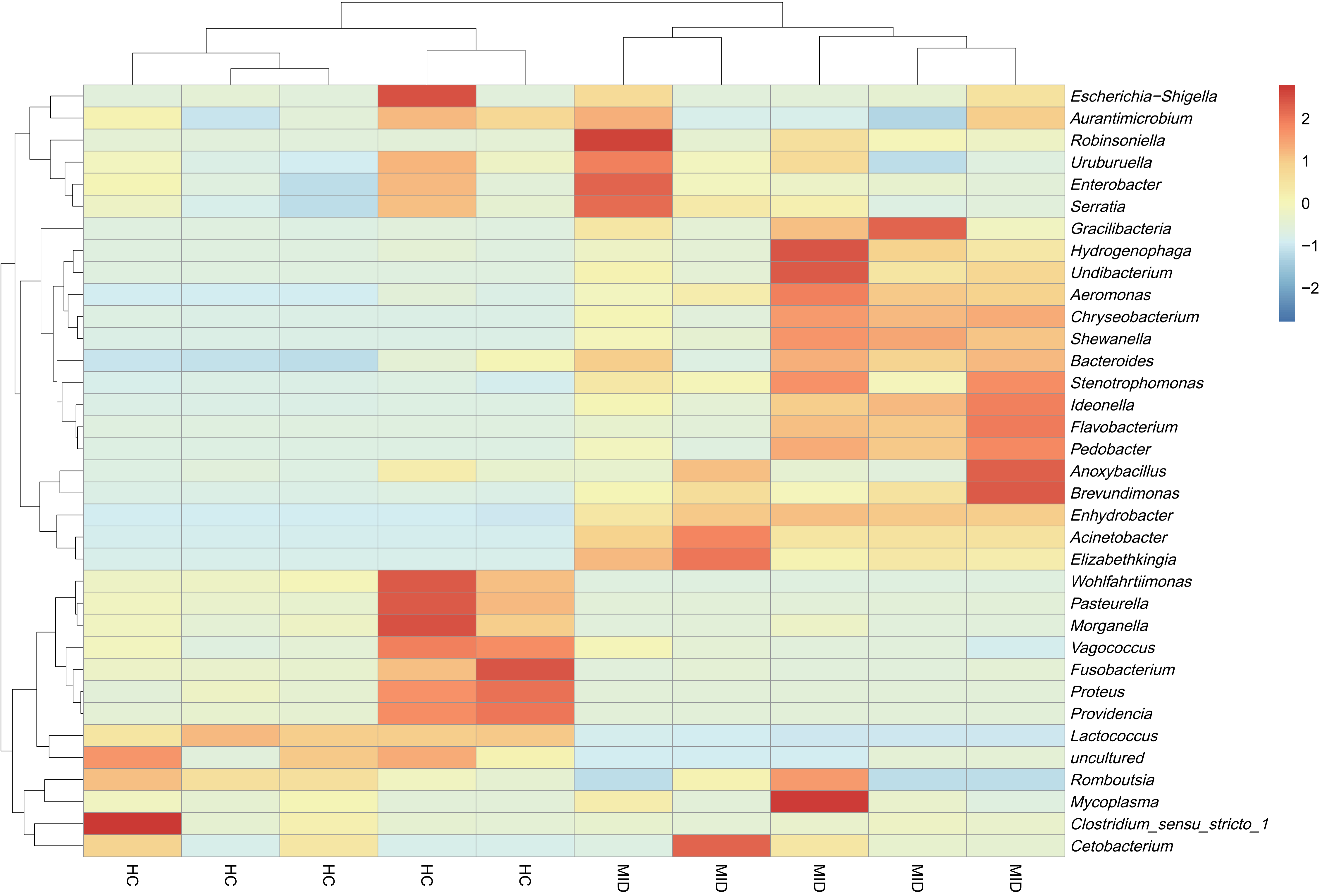

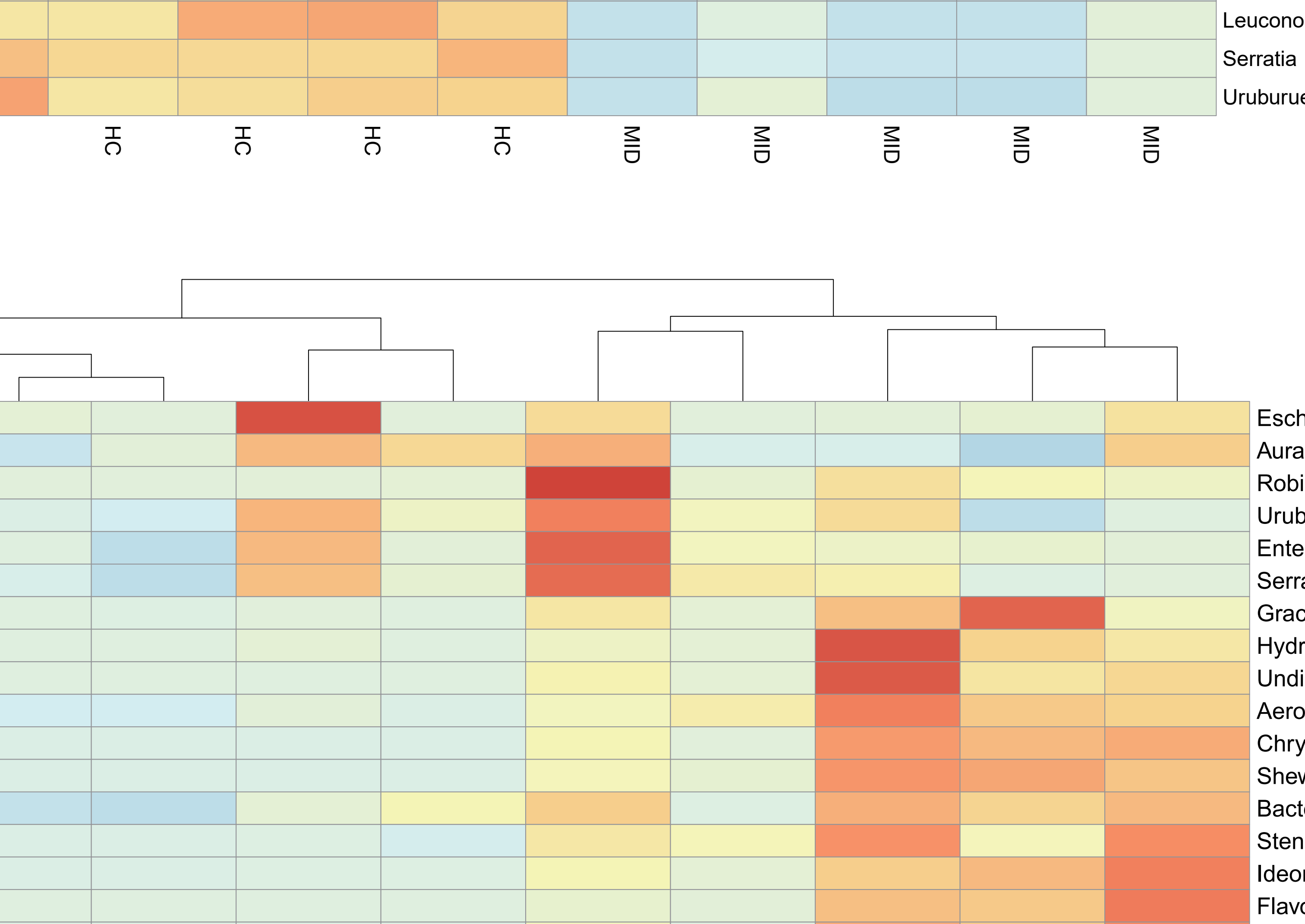

Supplement: Supplementary file 6 [file Data_Sheet_6.pdf]
