# Supplementary material for: Microbial community structure dynamics of invasive bullfrog with meningitis-like infectious disease
Source: Front Microbiol. 2023 Mar 13;14:1126195. doi: 10.3389/fmicb.2023.1126195 (PMC10040567; doi:10.3389/fmicb.2023.1126195)
Supplement: Supplementary file 7 [file Table_1.docx]

**Table 2 Basic data for sequencing oral microbiome samples**

| Group | Reads-raw | Reads-derep | **Average Reads-derep** |
| --- | --- | --- | --- |
| HC | 130389 | 79633 | 64549 |
|  | 104094 | 50821 |  |
|  | 98669 | 49365 |  |
|  | 133214 | 70968 |  |
|  | 153602 | 71960 |  |
| MID | 129252 | 83286 | 100243 |
|  | 195408 | 128809 |  |
|  | 179203 | 100140 |  |
|  | 143606 | 94194 |  |
|  | 145760 | 94784 |  |
