# Supplementary material for: Microbial community structure dynamics of invasive bullfrog with meningitis-like infectious disease
Source: Front Microbiol. 2023 Mar 13;14:1126195. doi: 10.3389/fmicb.2023.1126195 (PMC10040567; doi:10.3389/fmicb.2023.1126195)
Supplement: Supplementary file 8 [file Table_2.docx]

**Table 1 Basic data for sequencing gut microbiome samples**

| **Group** | **Reads-raw** | **Reads-derep** | **Average Reads-derep** |
| --- | --- | --- | --- |
| HC | 149161 | 121237 | 97876 |
|  | 92792 | 70279 |  |
|  | 115658 | 90554 |  |
|  | 126904 | 99338 |  |
|  | 140068 | 107973 |  |
| MID | 119440 | 59623 | 70080 |
|  | 96755 | 53670 |  |
|  | 167344 | 89733 |  |
|  | 99351 | 57747 |  |
|  | 112488 | 89625 |  |
